# Circulating pre-treatment Epstein-Barr virus DNA as prognostic factor in locally-advanced nasopharyngeal cancer in a non-endemic area

**DOI:** 10.18632/oncotarget.17822

**Published:** 2017-05-11

**Authors:** Salvatore Alfieri, Nicola Alessandro Iacovelli, Sara Marceglia, Irene Lasorsa, Carlo Resteghini, Francesca Taverna, Arabella Mazzocchi, Ester Orlandi, Marco Guzzo, Roberto Bianchi, Diana Fanti, Laura Pala, Sara Racca, Roee Dvir, Pasquale Quattrone, Annunziata Gloghini, Chiara Costanza Volpi, Roberta Granata, Cristiana Bergamini, Laura Locati, Lisa Licitra, Paolo Bossi

**Affiliations:** ^1^ Department of Medical Oncology 3, Fondazione IRCCS Istituto Nazionale dei Tumori, Milan, Italy; ^2^ Department of Radiation Oncology, Fondazione IRCCS Istituto Nazionale dei Tumori, Milan, Italy; ^3^ Department of Engineering and Architecture, University of Trieste, Trieste, Italy; ^4^ Laboratory of Immunohematology & Transfusion Medicine, Fondazione IRCCS Istituto Nazionale dei Tumori, Milan, Italy; ^5^ Department of Head and Neck Surgery, Fondazione IRCCS Istituto Nazionale dei Tumori, Milan, Italy; ^6^ Laboratory of Clinical Chemistry and Microbiology, ASST Grande Ospedale Metropolitano Niguarda, Milan, Italy; ^7^ Department of Medical Oncology of Melanoma and Sarcoma, Istituto Europeo di Oncologia, Milan, Italy; ^8^ Laboratory of Clinical Microbiology & Virology, San Raffaele IRCCS Hospital, Milan, Italy; ^9^ Department of Pathology, Fondazione IRCCS Istituto Nazionale dei Tumori, Milan, Italy; ^10^ Department of Medical Oncology 3, University of Milan, Milan, Italy

**Keywords:** nasopharyngeal cancer, Epstein-Barr virus, prognosis, head and neck cancer, non endemic

## Abstract

The prognostic value of pre-treatment Epstein-Barr Virus (EBV) DNA viral load for non-endemic, locally-advanced, EBV-related nasopharyngeal cancer (NPC) patients is yet to be defined. All patients with EBV encoded RNA (EBER)-positive NPC treated at our Institution from 2005 to 2014 with chemotherapy (CT) concurrent with radiation (RT) +/- induction chemotherapy (ICT) were retrospectively reviewed. Pre-treatment baseline plasma EBV DNA (b-EBV DNA) viral load was detected and quantified by PCR. Median b-EBV DNA value was correlated to potential influencing factors by univariate analysis. Significant variables were then extrapolated and included in a multivariate linear regression model. The same variables, including b-EBV DNA, were correlated with Disease Free Survival (DFS) and Overall Survival (OS) by univariate and multivariate analysis.

A total of 130 locally-advanced EBER positive NPC patients were evaluated. Overall, b-EBV DNA was detected in 103 patients (79.2%). Median viral load was 554 copies/mL (range 50–151075), and was positively correlated with T stage (p=0.002), N3a-b vs N0-1-2 stage (p=0.048), type of treatment (ICT followed by CTRT, p=0.006) and locoregional and/or distant disease recurrence (p=0.034). In the overall population, DFS and OS were significantly longer in patients with pre-treatment negative EBV DNA than in positive subjects at the multivariate analysis.

Negative b-EBV DNA can be considered as prognostic biomarker of longer DFS and OS in NPC in non-endemic areas. This finding needs confirmation in larger prospective series, with standardized and inter-laboratory harmonized method of plasma EBV DNA quantification.

## INTRODUCTION

Nasopharyngeal cancer (NPC), commonly affecting Asian countries (incidence rate as high as 20-50 per 100.000 persons/year), is a very rare disease in Caucasian countries (0.5 new cases/year per 100.000 persons/year) [1, 2]. Epstein-Barr Virus (EBV) is one of the most important causative factors of NPC. It has a heterogeneous diffusion, which might account, at least in part, for the different incidence between Southern China or Southeast Asia (endemic area) and Europe or USA (non-endemic area).

In the endemic area, the prognostic role of plasma EBV DNA viral load, with different cut-offs and detected before curative treatment for locally-advanced NPC patients, has been well described [3, 4, 5, 6].

To our knowledge, in the non-endemic area, only two limited case series (34 and 36 patients, respectively) investigating the role of EBV DNA have been reported [7, 8]. These studies showed a direct relationship between pre-treatment EBV DNA viral load and baseline clinical tumor stage, and between EBV DNA increase and tumor recurrence during follow-up. Ferrari et al. [8] tried to demonstrate a prognostic significance of pre-treatment EBV DNA by reporting a significant correlation between higher baseline EBV DNA values and shorter Disease-Free Survival (DFS) at univariate analysis (not confirmed after adjusting for age).

Therefore, the role of pre-treatment EBV DNA quantification for NPC patients in a non-endemic areas remains undefined. This study evaluates the prognostic role of baseline EBV DNA viral load for locally-advanced EBV-related NPC patients in a non-endemic area.

## RESULTS

### Study population

As shown in Table 1, 130 EBER-positive NPC patients were treated at our Institution with curative intent. The majority of patients were males (66.9%), in good clinical conditions (ECOG PS 0-1: 95.4%), young (median age: 48.5, range: 18-81 years), mainly staged as locally-advanced disease (86.2%: III-IV stage) and treated with induction chemotherapy before concomitant CTRT (77.7%: ICT with CTRT). One out of 5 patients was referred to our hospital after diagnostic neck surgery. The median b-EBV DNA viral load was 554 copies/mL with only 5 (3.8%) positive UQ and 27 (20.8%) negative patients. At a median follow-up of 43 months (range: 11-122) 28 patients recurred (21.5%) and 18 (13.9%) had died. The majority of recurrences (24/28; mean month: 37.9, median month: 38, range: 7-60 months) and all deaths (mean month: 40.9, median month: 43, range: 11-60 months) occurred within 5 years of follow-up.

**Table 1:** Baseline characteristics of study population and descriptive statistics

Characteristics	*N* (% or range)
Gender	
M	87 (66.9)
F	43 (33.1)
Median Age	48.5 (18-81)
ECOG Performance Status	
0-1	124 (95.4)
2	6 (4.6)
Treatment	
ICT followed by CTRT	101 (77.7)
CTRT	29 (22.3)
Neck surgery^a^	
YES	26 (20)
NO	104 (80)
Stage (VII AJCC)	
II	18 (13.8)
III	40 (30.8)
IVa	26 (20)
IVb	46 (35.4)
Median EBV DNA	554 (0-151075)
EBV DNA^b^	
UQ	5 (3.8)
Neg	27 (20.8)
Q	50 (38.5)
Q+	48 (36.9)
Relapse	**28 (21.5)**
Locoregional	12 (42.9)
Distant	16 (57.1)
Death	**18 (13.9)**
Due to disease	15 (83.3)
Due to other causes	3 (16.7)

ECOG: Eastern Cooperative Oncology Group.

AJCC: American Joint Committee on Cancer.

^a^ Lymph node neck dissection or excisional biopsy.

^b^ EBV DNA was stratified into 4 groups: Neg, Negative (EBV DNA = 0); UQ, Positive but UnQuantifiable (0 < EBV DNA < 10^2^ copies/mL); Q, Positive and quantifiable (10^2^ ≤ EBV DNA ≤ 15 × 10^2^ copies/mL); Q+, Strongly positive and quantifiable (EBV DNA > 15 × 10^2^ copies/mL).

### b-EBV DNA and its influencing factors

Median b-EBV DNA viral load was significantly higher (Table 2) in patients with: ICT followed by CTRT (*P*=0.006), higher tumor T and N stage (T4 and N3a-3b, *P*=0.002 and *P*=0.048), locoregional and/or distant disease recurrence (*P*=0.034).

**Table 2:** Univariate analysis of b-EBV DNA and its influencing factors

Characteristics	EBV DNA(median)^a^	*P* value^b^
**Gender**		
M	545	0.094
F	577	
**ECOG Performance Status**		
0-1	549.5	0.112
2	6820	
**Treatment**		
ICT followed by CTRT	885	**0.006**
CTRT	179	
**Neck surgery^c^**		
YES	835	0.083
NO	226.5	
**T classification**		
1-2-3	387	**0.002**
4	1745	
**N classification**		
0-1-2	453	**0.048**
3a-3b	2153	
**Recurrence**		
YES	4609	**0.034**
NO	480	

^a^ Excluded from this analysis the Unquantifiable EBV DNA group (*N*=5).

^b^ Continuous variable (*age*) was assessed using Spearman's correlation; categorical variables were assessed using non-parametric Mann-Whitney test. Statistically significant *P* values (*P*<0.05) are highlighted.

^c^ Lymph node neck dissection or excisional biopsy.

### b-EBV DNA and outcome variables (DFS, OS)

**Figure 1:**
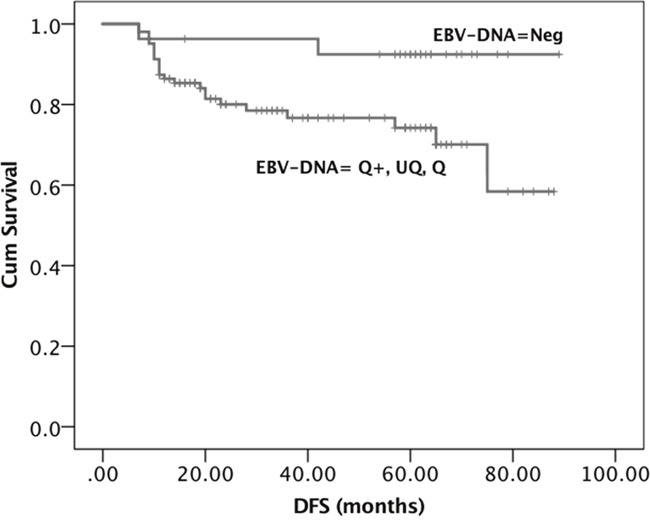
Kaplan-Meier survival curves showing the probability of DFS in locally advanced EBER positive NPC patients DFS= Disease Free Survival. EBV DNA was stratified into 4 groups: Neg, Negative (EBV DNA = 0); UQ, Positive but UnQuantifiable (0 < EBV DNA < 10^2^ copies/mL); Q, Positive and quantifiable (10^2^ ≤ EBV DNA ≤ 15 × 10^2^ copies/mL); Q+, Strongly positive and quantifiable (EBV DNA > 15 × 10^2^ copies/mL).

**Figure 2:**
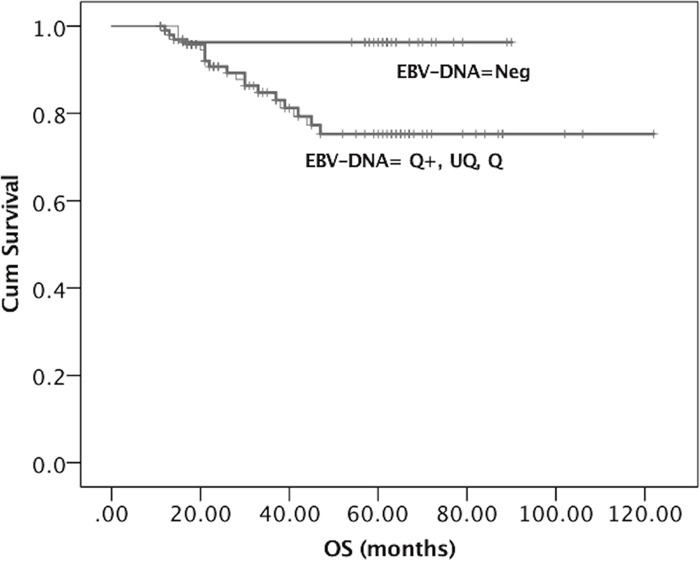
Kaplan-Meier survival curves showing the probability of OS in locally advanced EBER positive NPC patients OS= Overall Survival. EBV DNA was stratified into 4 groups: Neg, Negative (EBV DNA = 0); UQ, Positive but UnQuantifiable (0 < EBV DNA < 10^2^ copies/mL); Q, Positive and quantifiable (10^2^ ≤ EBV DNA ≤ 15 × 10^2^ copies/mL); Q+, Strongly positive and quantifiable (EBV DNA > 15 × 10^2^ copies/mL).

At univariate analysis, DFS was significantly longer in females (*P*=0.035), patients who underwent neck surgery (*P*=0.035), and those with lower T-stage (T1-2-3, *P*=0.006) and negative b-EBV DNA values (*P*=0.002). OS was significantly longer in low T-staged (T1-2-3, *P*=0.018) and negative b-EBV DNA NPC patients (*P*=0.027). At multivariate analysis, only b-EBV DNA negativity (vs positivity) was confirmed as significantly independent influencing factor for both DFS and OS (*P*=0.05 and *P*=0.06, respectively). Kaplan-Meier survival curves (Figures 1, 2) showed a significant relationship between b-EBV DNA status (negative vs positive) and outcome variables (DFS, *P*=0.032 and OS, *P*=0.029).

### Identification of b-EBV DNA cut-off influencing outcome

**Figure 3:**
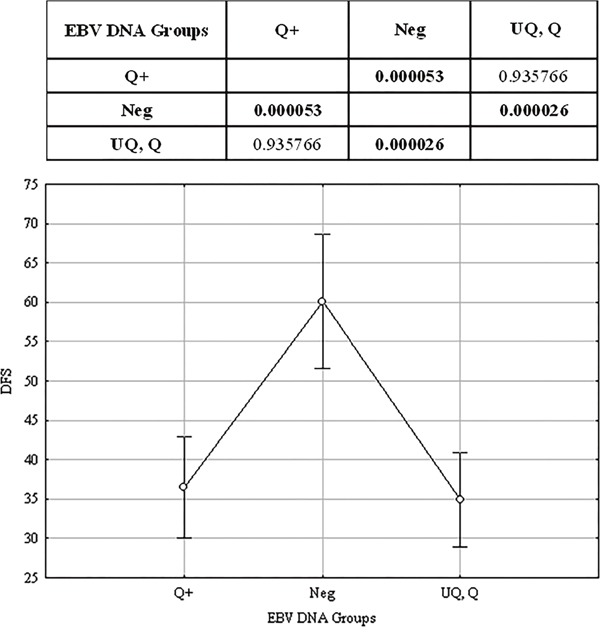
Results of Tukey HSD test EBV DNA was stratified into 4 groups: Neg, Negative (EBV DNA = 0); UQ, Positive but UnQuantifiable (0 < EBV DNA < 10^2^ copies/mL); Q, Positive and quantifiable (10^2^ ≤ EBV DNA ≤ 15 × 10^2^ copies/mL); Q+, Strongly positive and quantifiable (EBV DNA > 15 × 10^2^ copies/mL). The upper panel shows the results of the Tukey's HSD test: *P* values corresponding to the difference between group pairs are displayed. Highlighted values are statistically significant (*P*<0.05). The lower panel shows the median values of the three groups and their confidence intervals.

Considering b-EBV DNA as a categorical variable stratified into the 4 groups (Table 3), we disclosed a significant relationship between b-EBV DNA and DFS/OS. The UQ, Q and Q+ b-EBV DNA groups showed similar results, thus suggesting that UQ behave as Q and Q+. In order to verify this hypothesis, we re-run the test after grouping together UQ and Q. The results were the same (Table 3) and the relationship between b-EBV DNA and DFS and OS remained significant (*P*<0.001), like when UQ and Q were considered separately. Using these three groups (UQ and Q, Q+, and Neg), we found that the UQ and Q group had the same behaviour as the Q+ group, whereas both differed from the Neg (Figure 3), thus suggesting that b-EBV DNA can be considered as a binary variable (positive or negative) regardless of the number of copies measured.

**Table 3:** Univariate analysis of b-EBV DNA and outcome variables (DFS, OS)

EBV DNA^a^	DFS(median)	*P* value^b^	OS(median)	*P* value^b^
**4 Groups**				
UQ	13		13	
Neg	62	**0.0001**	62	**0.0001**
Q	27		31	
Q+	33.5		37.5	
**3 Groups**				
UQ, Q	23		26	
Q+	33.5	**0.0001**	37.5	**0.0002**
Neg	62	62		

^a^ EBV DNA was stratified into 4 and 3 groups: Neg, Negative (EBV DNA = 0); UQ, Positive but UnQuantifiable (0 < EBV DNA < 10^2^ copies/mL); Q, Positive and quantifiable (10^2^ ≤ EBV DNA ≤ 15 × 10^2^ copies/mL); Q+, Strongly positive and quantifiable (EBV DNA > 15 × 10^2^ copies/mL).

^b^ The categorical variables were assessed using non-parametric Kruskal Wallis ANOVA. Statistically significant *P* values (*P*<0.05) are highlighted.

**Figure 4:**
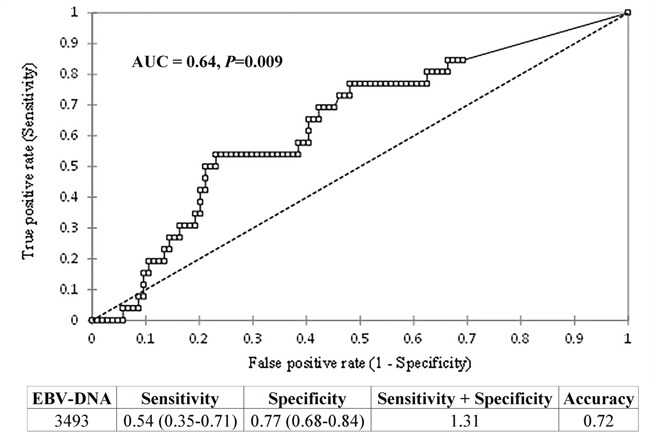
Results of ROC curve analysis for the individuation of a possible value of b-EBV DNA as cut-off of higher risk of locoregional/distant recurrence Top panel: ROC curve. The x-axis is (1-Specificity); the y-axis is Sensitivity, Area Under the Curve (AUC) = 0.64, *P*=0.009. Bottom panel: values of Specificity, Sensitivity, Accuracy, and Specificity+Sensitivity corresponding to the cut-off value of 3493 copies/mL. The lower and upper confidence intervals for Specificity and Sensitivity are reported in brackets.

By ROC curve analysis (Area Under the Curve, AUC=0.640, *P*=0.009) a b-EBV DNA value of 3493 copies/mL (Figure 4) was identified as cut-off of higher risk for loco-regional and/or distant recurrence with an accuracy of 72% (sensitivity: 53%; specificity: 76%).

### b-EBV DNA and outcome variables (DFS, OS) in a filtered-treatment study population

The median b-EBV DNA value was 885 copies/mL (range: 0 – 151075) in a filtered-treatment study population including only patients treated with ICT followed by CTRT (*N*=101). According to the results obtained in the overall population, b-EBV DNA was considered as a binary variable (positive/negative) and was significantly related with DFS (*P*=0.004) and OS (*P*=0.019) at univariate analysis.

Figures related to Kaplan-Meier survival curves (Supplementary Figure 1, Supplementary Figure 2) and the Tukey HSD post-hoc analysis results (Supplementary Figure 3) of the filtered-treatment study population are available as supplementary materials.

## DISCUSSION

We showed the prognostic value of pre-treatment plasma EBV DNA viral load in a relatively high number of non-endemic EBV-related NPC patients. To date, all data regarding this issue derived from the main clinical trials conducted in the endemic area [3, 4, 6].

The median copy number of b-EBV DNA (554 copies/mL) and the prevalence of positivity (79.2%) of plasma EBV DNA were quite lower than in the endemic area (median copy number: 681 for stage III, 1703 for stage IV; percentage of positivity: 94.9%) [3].

The median b-EBV DNA value resulted significantly higher in patients with more advanced disease stage and/or treated with ICT. The former observation reflects the correlation with the disease burden [9, 10, 11, 12, 13]; the latter may be explained by the fact that, during the entire time of our analysis, one of the criteria we adopted as Institutional policy for choosing ICT was b-EBV DNA higher than 15×10^2^ copies/mL as well as T4 and/or N3 disease.

At multivariate analysis, b-EBV DNA value correlated with both recurrence of disease and survival outcomes (DFS, OS), overcoming the variable of ICT.

Differing from what observed in endemic NPC populations [3, 4, 5, 6], we were not able to identify any cut-off of b-EBV DNA as influencing DFS and OS. The few (*N*=5) patients with unquantifiable positivity of b-EBV DNA, showed a similar behaviour as all other detectable b-EBV DNA groups thus supporting the informative baseline power of the test.

Our ROC curve analysis identified in 3493 copies/mL a cut-off associated with an increased risk of loco-regional and/or distant disease recurrence confirming in non-endemic area a trend of correlation between b-EBV DNA values progressively rising over the upper normal limits and recurrence.

This is in line with the results shown in an endemic population by Leung on 2003 [5] and Chen on 2016 [6]. They both found a cut-off of 4000 copies/mL for higher risk of development of distant metastases, which is one of the most important causes of treatment failure.

The main limitation of our analysis is the use of a laboratory method to measure circulating EBV DNA different from that used in the most robust published studies establishing the prognostic value of EBV DNA in NPC [3, 4], in which the primer/probe sets as target the BamHI-W region of the EBV genome [14]. *Bam*HI-W fragment is repeated 8 to 11 times in the EBV genome, thus allowing more sensitive detection when compared with a single copy EBV genes [15, 16], such as Latent Membrane Protein (LMP2), Polymerase1 (POL1) or Epstein-Barr Virus Nuclear Antigen 1 (EBNA-1). This latter was used in this study and may explain why we identified a category (the UQ group) that has never been reported before. Possibly, a higher-sensible methodology could have moved this UQ category to the quantifiable group. Moreover, these discrepancies of the laboratory evaluations may be the reasons why we did not find a specific cut-off to significantly impact on outcome. Furthermore, the use of this different laboratory method could also explain why we detected a lower prevalence of positivity and median copy number of EBV DNA compared with endemic populations; to this respect, however, we cannot rule out that genetic factors (i.e., different genetic profile between Asian and non-Asian populations) have played a role.

On the other hand, the EBV DNA detection method targeting *Bam*HI-W, which is a very variously repeated region among all EBV strains genomes, could be linked to a higher inter-subjects variability rate.

The commutability of all EBV DNA values across all different methods is a very debated issue [17, 18]. Le QT et al. [19] already stated that a prompt assay standardization is mandatory to reduce the intra and inter-laboratory variability to ensure a better comparison of all worldwide collected plasma EBV DNA samples.

Another limitation of our analysis is the non-homogeneous characteristic of the retrieved data. The main imbalance was due the high rate of patients having performed an ICT before locoregional treatment. However, we also demonstrated that this variable did not influence the prognostic value of b-EBV DNA.

Another weakness of our study is the lack of information about the post-treatment EBV DNA viral load detection which has already been recognized in endemic area as significantly related to worse prognosis when still detectable for 1 week [3, 4, 20] to 1 month-time after chemo-radiotherapy completion [21]. Noteworthy, two ongoing randomized, phase III trials (NCT00370890 and NCT02363400) will evaluate NPC patients with residual EBV DNA to adjuvant chemotherapy or clinical observation only. The results of those trials will help to understand if post-treatment EBV DNA is the best factor for identifying patients more likely to benefit of the adjunctive therapies.

However, based on our findings that suggest a prognostic value of pre-treatment EBV DNA in patients belonging to non-endemic areas, we advocate for international cooperation to allow for standardized and inter-laboratory harmonized method of plasma EBV detection. A routinary and comparable quantification of EBV DNA may be of relevance for a timely monitoring of disease even in other settings, such as the follow-up phase and metastatic stage.

## MATERIALS AND METHODS

We evaluated the pre-treatment baseline plasma EBV DNA (b-EBV) analysed in all EBV positive NPC patients treated with curative intent at the National Cancer Institute of Milan – a referral Center for the treatment of oncological disease in Northern Italy - from March 2005 to May 2014. EBV infection was determined by EBV encoded RNAs (EBER) *in situ* hybridization.

### EBV DNA detection

EBV DNA levels were measured on plasma samples by real-time quantitative polymerase chain reaction (q-PCR) and were expressed as copies/mL. This measurement was performed at two Hospitals of Milan, Niguarda Ca’ Granda Hospital and San Raffaele Hospital. Viral DNA extraction was performed by using the NucliSENS easyMAG kit (BioMérieux, Lyon, France) in Niguarda Hospital and Qiagen MDX kit in San Raffaele Hospital. In both Hospitals, EBV DNA quantification was performed by EBV Elite MGB kit according to the manifacturer's instructions. The system amplified the gene coding for Epstein-Barr Virus Nuclear Antigen 1 (EBNA-1) protein.

The linear range of the assay was 10^2^-10^7^ copies/mL and the results of EBV DNA were expressed as follows: Not detected; Detected and Quantifiable (≥10^2^ copies/mL); Detected but UnQuantifiable [UQ] (< 10^2^ copies/mL).

### Oncologic treatment

Patients were treated with chemo-radiotherapy (CTRT) with or without induction chemotherapy (ICT) according to the standard practice of our Institute.

### Radiotherapy

All patients were treated with intensity modulated radiotherapy (RT) techniques (IMRT, Intensity Modulated Radiation Therapy or VMAT, Volumetric Modulated Arc Therapy), with a curative intent. In all patients, total prescription dose was 70 Gy, either by conventional fractionation (2 Gy per fraction, 5 fractions per week) or a moderately-hypofractionated regimen (2.12 Gy per fraction, 5 fractions per week), using a sequential or simultaneous integrated boost approach. All patients were staged with MRI of the head and neck district and whole body PET-CT scan before the initiation of oncological therapy; all images were fused with planning CT scans to better define target volumes. In patients receiving ICT, the extent of disease was re-evaluated with MRI after the last chemotherapy cycle. Planning procedures were usually performed shortly after the completion of ICT, and RT usually started within 3 to 4 weeks from the last cycle of induction chemotherapy.

### Chemotherapy

In line with Institutional policies, ICT was prescribed in patients with: a) clinically-staged T4 and/or N3a-b NPC; b) Neck surgery (lymph node neck dissection or excisional biopsy) performed for diagnostic purposes; c) b-EBV DNA higher than 15×10^2^ copies/mL according to the strongest dismal prognostic cut-off of b-EBV DNA recognized by Lin et al. [3]. In more details, Lin et al. showed that endemic NPC patients with baseline plasma EBV DNA concentrations < 1500 copies/mL had better outcomes (in terms of OS and DFS) compared with those with pre-treatment EBV DNA values ≥1500 copies/mL.

ICT was administered with TPF schedule for 3 cycles every 3 weeks (docetaxel 75 mg/sm on day 1, cisplatin 75 mg/sm on day 1, 5-fluororuracil 750 mg/sm/day on days 1 to 4) followed by antibiotic prophylaxis (ciprofloxacin at 500 mg dose twice a day as standard) from 5^th^ to 15^th^ day after chemotherapy initiation. The use of growth-stem cell factors (G-CSF) as primary prevention for chemo-related neutropenia was limited to patients with intracranial disease or at high-risk of infection development. Concomitant chemotherapy regimen was weekly (50 mg/sm) or 3-weekly (100 mg/sm) cisplatin (carboplatin AUC 5 was preferred if baseline creatinine clearance was lower than 60 ml/min).

### Statistical analysis

Descriptive statistics were calculated over main baseline patients [gender, age, Eastern Cooperative Oncology Group Performance Status (ECOG PS)], disease (T stage, N stage, VII AJCC Stage, locoregional and/or distant recurrence) and treatment characteristics (CTRT, CTRT with ICT, Neck Surgery).

We first investigated the relationship of b-EBV DNA viral load with all patients, disease, and treatment-related characteristics that could be considered influencing factors.

Univariate analysis was performed: non-parametric Mann-Whitney test (Gender, ECOG PS, CTRT vs CTRT with ICT, Neck Surgery) or non-parametric Kruskal Wallis ANOVA (T stage, N stage) was used for categorical variables (*P*<0.05). Spearman's correlation (Age) was used for continuous variables (*P*<0.05). Subjects with positive UQ EBV DNA value were excluded from this analysis; patients with T stage 1, 2, and 3 were grouped together and compared with patients with T stage 4, while patients with N stage 0, 1, and 2 were grouped together and compared with patients with N stage 3a and 3b.

Second, we investigated the relationship of outcome variables (DFS, OS) with the above-mentioned influencing factors and with b-EBV DNA. To this end, EBV DNA was stratified into 4 groups:

1. Negative (Neg): b-EBV DNA = 0 copies/mL;
2. Positive but UnQuantifiable (UQ): 0 < b-EBV DNA <10^2^ copies/mL;
3. Positive and quantifiable (Q): 10^2^ ≤ b-EBV DNA ≤15×10^2^ copies/mL;

Strongly positive and quantifiable (Q+): b-EBV DNA >15×10^2^ copies/mL.

A cut-off of 15×10^2^ copies/mL was chosen in accordance with existing evidence [3], as previously mentioned. Tukey's Honest test was used for post-hoc analysis (*P*<0.05).

Survival analysis (Kaplan-Meier) was conducted on OS and DFS with respect to all the variables that resulted significant at the univariate analysis.

In order to verify our results in a homogenously treated cohort of patients, we considered separately the subgroup of patients treated with ICT followed by CTRT or with CTRT alone. The same analysis described for the entire population was conducted in this subgroup. Last, a ROC analysis was performed on EBV DNA values in order to establish the threshold value that might better discriminate patients with recurrence from patients without recurrence.

Provision of study materials or patients: Pasquale Quattrone, Annunziata Gloghini, Chiara Costanza Volpi, Diana Fanti, Sara Racca, Roee Dvir, Marco Guzzo, Roberto Bianchi, Ester Orlandi, Lisa Licitra.

Collection and assembly of data: Salvatore Alfieri, Carlo Resteghini, Laura Pala, Nicola Alessandro Iacovelli, Cristiana Bergamini, Roberta Granata.

Data analysis and interpretation: Salvatore Alfieri, Paolo Bossi, Sara Marceglia, Irene Lasorsa, Laura Locati, Lisa Licitra.

Manuscript writing: Salvatore Alfieri, Paolo Bossi, Sara Marceglia, Irene Lasorsa, Nicola Alessandro Iacovelli, Francesca Taverna, Arabella Mazzocchi, Diana Fanti.

Final approval of manuscript: All authors.

## SUPPLEMENTARY MATERIALS FIGURES


