# Supplementary material for: Circulating pre-treatment Epstein-Barr virus DNA as prognostic factor in locally-advanced nasopharyngeal cancer in a non-endemic area
Source: Oncotarget. 2017 May 11;8(29):47780–9. doi: 10.18632/oncotarget.17822 (PMC5564604; doi:10.18632/oncotarget.17822)
Supplement: Supplementary file 1 [file oncotarget-08-47780-s001.pdf]

Circulating pre-treatment Epstein-Barr virus DNA as prognostic factor in locally-advanced nasopharyngeal cancer in a non-endemic area

SUPPLEMENTARY FIGURES

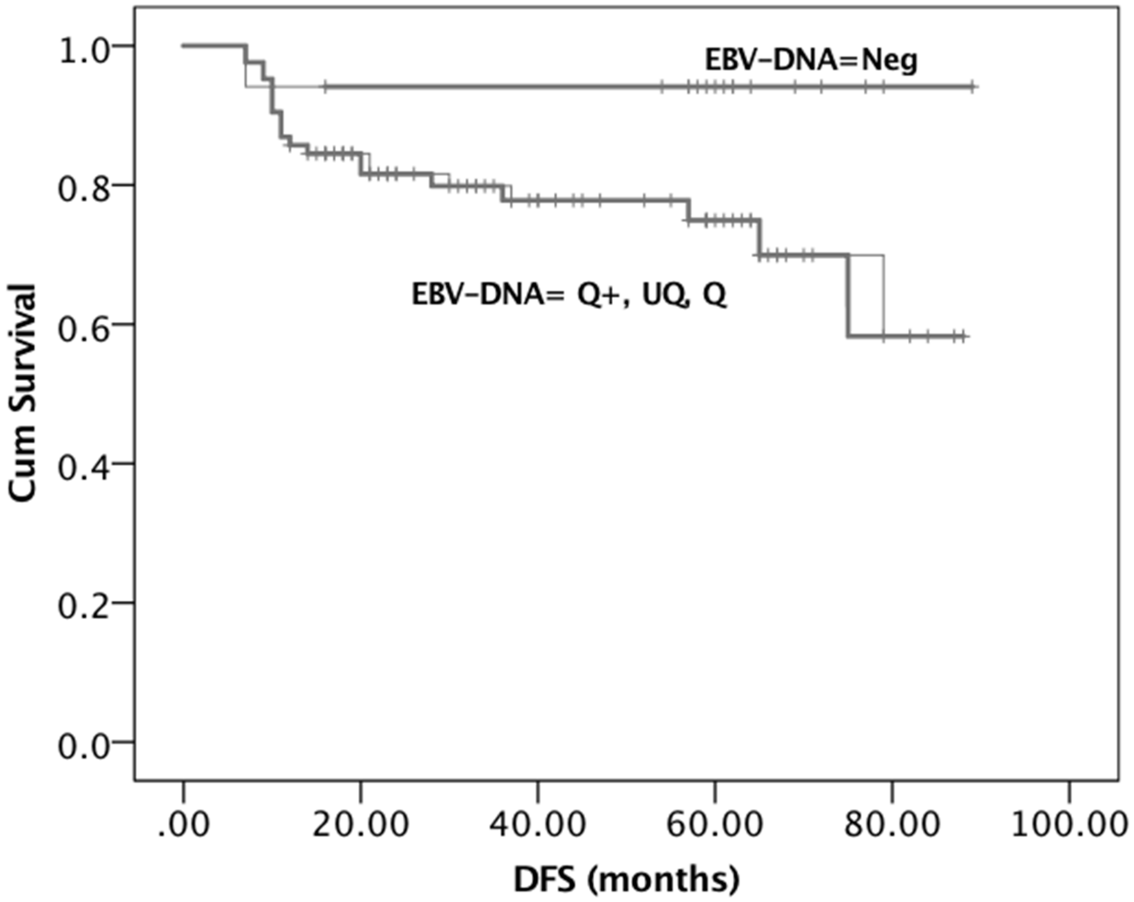

Supplementary Figure 1: Kaplan-Meier survival curves showing the probability of DFS in locally advanced EBER positive NPC patients, post-hoc analysis. DFS= Disease Free Survival. EBV DNA was stratified into 4 groups: Neg, Negative (EBV DNA = 0); UQ, Positive but UnQuantifiable (0 < EBV DNA < 10^2 copies/mL); Q, Positive and quantifiable ($10^2 \leq$ EBV DNA $\leq 15 \times 10^2$ copies/mL); Q+, Strongly positive and quantifiable (EBV DNA > 15×10^2 copies/mL).

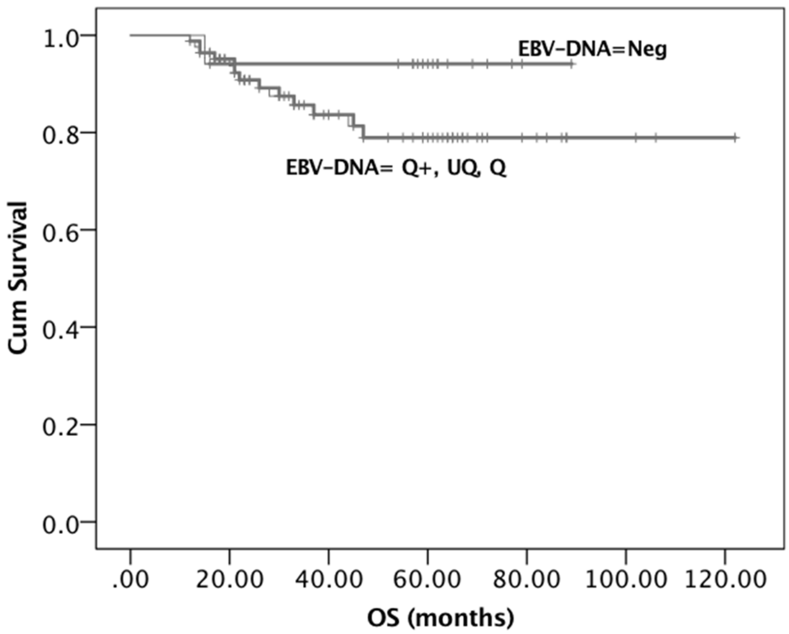

Supplementary Figure 2: Kaplan-Meier survival curves showing the probability of OS in locally advanced EBER positive NPC patients, post-hoc analysis. OS= Overall Survival. EBV DNA was stratified into 4 groups: Neg, Negative (EBV DNA = 0); UQ, Positive but UnQuantifiable ($0 < \text{EBV DNA} < 10^2$ copies/mL); Q, Positive and quantifiable ($10^2 \leq \text{EBV DNA} \leq 15 \times 10^2$ copies/mL); Q+, Strongly positive and quantifiable ($\text{EBV DNA} > 15 \times 10^2$ copies/mL).

EBV DNA Groups	Q+	Neg	UQ, Q
Q+		**0.002907**	0.847343
Neg	**0.002907**		**0.010824**
UQ, Q	0.847343	**0.010824**	

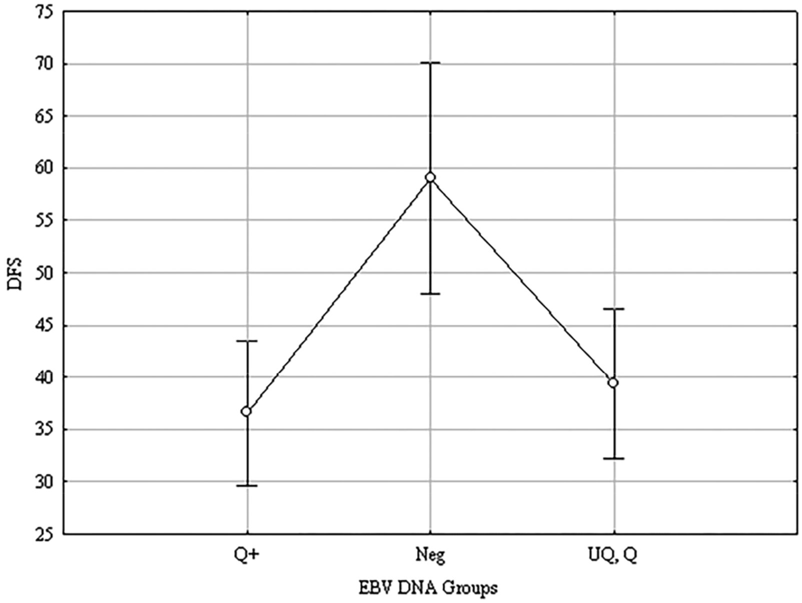

Supplementary Figure 3: Results of Tukey HSD test, post-hoc analysis. EBV DNA was stratified into 4 groups: Neg, Negative (EBV DNA = 0); UQ, Positive but UnQuantifiable (0 < EBV DNA < 10^2 copies/mL); Q, Positive and quantifiable ($10^2 \leq$ EBV DNA $\leq 15 \times 10^2$ copies/mL); Q+, Strongly positive and quantifiable (EBV DNA > 15×10^2 copies/mL). The upper panel shows the results of the Tukey's HSD test: *P* values corresponding to the difference between group pairs are displayed. Highlighted values are statistically significant ($P<0.05$). The lower panel shows the median values of the three groups and their confidence intervals.
